# A Fuzzy Logic-Based Cost Modelling System for Recycling Carbon Fibre Reinforced Composites

**DOI:** 10.3390/polym13244370

**Published:** 2021-12-14

**Authors:** Essam Shehab, Arshyn Meiirbekov, Akniyet Amantayeva, Aidar Suleimen, Serik Tokbolat, Shoaib Sarfraz, Md Hazrat Ali

**Affiliations:** 1 Mechanical and Aerospace Engineering Department, School of Engineering and Digital Sciences, Nazarbayev University, Nur-Sultan 010000, Kazakhstan; arshyn.meiirbekov@nu.edu.kz (A.M.); akniyet.amantayeva@nu.edu.kz (A.A.); aidar.suleimen@nu.edu.kz (A.S.); md.ali@nu.edu.kz (M.H.A.); 2 School of Architecture, Design and the Built Environment, Nottingham Trent University, Nottingham NG1 4FQ, UK; serik.tokbolat@ntu.ac.uk; 3 Design, Manufacturing and Production Engineering Section, Department of Mechanical and Production Engineering, Aarhus University, 8000 Aarhus, Denmark; ssarfraz@mpe.au.dk

**Keywords:** fuzzy logic, cost engineering, CFRP recycling techniques

## Abstract

Carbon Fibre Reinforced Polymers (CFRPs) are commonly used materials in manufacturing components and products in the automotive, aerospace, and wind energy industries generating thousands of tons of waste, thus creating a threat to the environment if not recycled. Therefore, it is important for both academia and industry to investigate various ways of recycling this material. However, there is an urgent need for a reliable cost predication system to assist in making informed decisions, planning sustainable treatment, and developing pricing strategies for different waste treatment scenarios. This research paper presents the development of a fuzzy logic-based system to perform cost estimation of recycling processes of the CFRP. The developed system has taken into consideration uncertainties such as the characteristics of End of Life (EoL) material including its size and weight, its origin and diversity of existing recycling methods, and quantity of recycling waste. Cost drivers were divided into categories such as dismantling, transportation, operation, and capital cost. The system was developed by creating 243 fuzzy rules and three levels of fuzzy sets. Moreover, an interactive user-friendly interface was developed to enable the user to use the system easily and efficiently. Finally, case study results were examined to compare the whole life recycling cost of four different recycling technologies in various scenarios of waste treatment. The developed fuzzy logic-based system has the capability in evaluating the cost structure of CFRP recycling techniques and take into consideration uncertainty factors. Hence, a major contribution of the developed system is its provision of the heuristic rules that aid the decision-making process for selecting a cost-effective recycling method. The visualisation facility of the developed system is also a useful tool in enabling potential users to forecast the cost of the CFRP recycling techniques upfront.

## 1. Introduction

Carbon Fibre Reinforced Polymers (CFRP) were first applied in the aerospace industry but later paved their way to other industries such as automotive, wind energy, and sports and leisure. The wide range of applications of these materials became possible due to their mechanical and chemical properties such as strength, elasticity, and lightweight. The global demand for carbon fibres (CF) is predicted to reach almost 200,000 tons in 2022 [1]. Materials used for aircraft components and wind turbine blades have about 25–50 years of useful life and then need to be disposed of. The current disposing methods are predominantly landfilling and incineration [2]. However, considering the increasing trend of composite materials and products manufacturing, rising environmental awareness, and legislative requirements, sustainable disposal methods require urgent attention.

Sustainability-related challenges arising from CFRP disposal such as landfill saturation, environmental pollution, and economic waste are becoming a significant problem around the world [3]. However, at present, only developed countries are introducing and implementing legal standards related to end-of-life disposal of CFRP waste [4]. Moreover, recycling mechanisms at a commercial scale are only available in a few countries such as the USA, Italy, UK, and Japan [5]. At present, the respective industries related stakeholders still tend to hesitate in utilizing recycled materials and neglect recycling as an end-of-life treatment [6]. However, close collaboration and information dissemination is required to promote CFRP recycling solutions to make them attractive to both waste holders and manufacturers.

There are limited research efforts on the economic and environmental feasibility of CFRP recycling. The main challenge is that there is a lack of enough data to develop cost estimation of CFRP recycling. Vo Dong et al. [7] studied lifecycle-based cost estimation of CFRP recycling pathways. However, the authors make several assumptions and do not consider other factors including the end of life (EoL) waste parameters, transportation, and dismantling. This indicates that the output results do not consider underlying uncertainty factors pertinent to recycling operations.

The financial feasibility of CFRP recycling depends on many input variables which are unique for every recycling method. The most commonly used recycling methods are mechanical, thermal (pyrolysis), and chemical (solvolysis) processes [8,9,10,11,12]. These methods have their own cost drivers which are associated with certain levels of uncertainty such as utilities and initial investments [13]. The prior research within the scope of this project included the exploration of cost drivers, challenges in estimating their uncertainties, and building cost estimation framework for recycling CFRPs [14,15,16]. This study adopted fuzzy logic to address the uncertainty factors in the cost estimation process of recycling CFRPs. The fuzzy set theory allows approximating the exact value based on predefined linguistic variables and fuzzy rules [17]. 

A fuzzy logic approach is appropriate when the data is not enough for constructing cost-estimating relationships (CERs) using regular approaches [18]. CERs are mathematical models to predict cost of a product or service using an established relationship with independent variables. The parameters which define the characteristics of the process are called cost drivers [19]. The cost drivers are linked to cost via CERs. The application of fuzzy logic for estimating the cost in various industrial processes and consumer products settings was found suitable due to its relatively simple nature not requiring complex mathematical models [19]. Historically, the initial fuzzy logic cost estimation models were developed at the end of the 20th century as shown in the work of Wiehn et al. [20] in which incineration plant cost with uncertain elements were modelled and expressed linguistically. Another work by Chansaad et al. [21] solved the problem of uncertainty of paint loss by developing a fuzzy logic-based cost estimation method for painting products with different geometric parameters. However, there were no studies found associated directly with estimating cost of recycling by implementing fuzzy elements. There are several works that were specifically related to recycling. For example, Phillis et al. [22] developed a method to assess material recyclability with the help of a multistage fuzzy inference process. The complex system with multistage inputs derives a measure of recyclability for any material based on available data. Another work by Keivanpour et al. [23] proposed a fuzzy logic-based system to assess the economic feasibility of end-of-life vehicle (ELV) dismantling under uncertain conditions. The authors were able to incorporate uncertainty and typically considered as uniform details of ELVs such as size, complexity, differences in models, and design to conduct a more accurate cost-benefit analysis of the process. Some key works studied the financial feasibility of CFRP recycling processes [7,24,25,26]; however, none of them dealt with the uncertainty in the estimation by implementing fuzzy values. 

The above literature indicated that there are no research efforts in predicting cost of recycling CFRP. It is also apparent that previous research studies did not take uncertainties of various stages of the CFRP recycling into consideration. Therefore, a fuzzy logic-based cost modelling system is presented in this paper. Cost of different CFRP recycling methods were compared with the currently applied landfilling charges to evaluate the financial attractiveness of recycling.

## 2. CFRP Recycling Cost Structure

This study proposed a framework for assessing cost of CFRP recycling considering the uncertainties inherent to the recycling process. The uncertainty factors associated with the recycling process were used as input parameters to a multistage fuzzy inference process. This process calculates the total cost of the recycling process based on selected parameters. The recycling processes considered in this work include mechanical recycling, pyrolysis, fluidized bed process (FBP), and solvolysis in supercritical water. 

**Figure 1:**
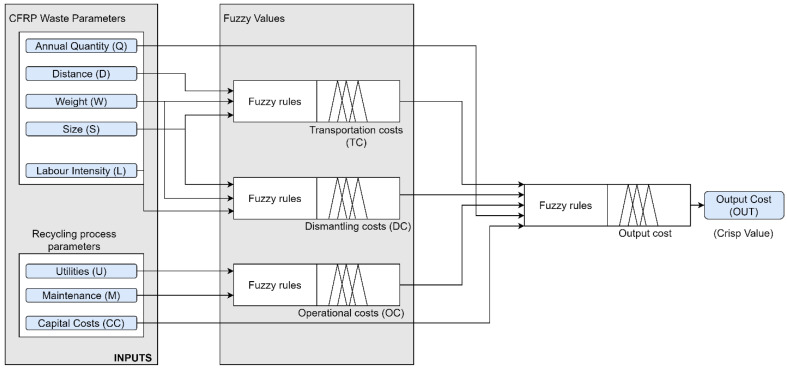
Architecture of the overall structure of the developed cost estimation system.

The input parameters in the developed system were divided into two categories. The first category of input parameters consists of data on the CFRP waste (weight, size, labour intensity), transportation distance, annual quantity required to recycle. The second category includes recycling technique-dependent parameters such as utility consumption levels, capital cost requirements. These parameters were converted to fuzzy sets and were processed in fuzzy inference engines represented in Figure 1. It is important to note that the inference system is multistage: first-stage inputs for intermediate inference engines are distance between the CFRP waste and recycling factory, waste weight and size, labour requirement, factory utilities and maintenance plan. Whereas second-stage inputs capital cost, annual quantity, transportation, dismantling, and operational cost are defined as inputs directly passing to output cost inference engine. Finally, the final output cost is converted to a crisp value. This section describes input cost parameters and provides a detailed explanation of fuzzy ranges.

### 2.1. Transportation Cost

Transportation cost comprises three input parameters such as *distance* between the recycling site and waste source, *weight*, and *size (volume)* of the transported waste parts. The weight and size of the CFRP waste vary from industry to industry, e.g., automotive composite parts and aircraft CFRP elements [27,28]. Therefore, the range of weights is defined within each industry. For example, in the wind energy industry, the average capacity of wind turbines worldwide is 1.39 MW, while each 1 kW corresponds to 10 kg of rotor blade weight (only 6% of which is CFRP) [3,29]. Thus, the medium-range weight for wind turbine blades (all three blades) is estimated to be around 13,900 kg (for all three blades). The wind turbine with 1.39 MW capacity is assumed to have 30 m long turbine blade with 50 m^3^ of volume [30]. Based on the weight and size of the waste, it is then decided how many trucks or other modes of transport are needed to transport it to the recycling site. For example, in Ireland, the wind turbine blade waste is cut into 1.5 m^2^ sized pieces and loaded by 6–8 tones to a lorry [31]. The range of the volumes is defined based on the necessity of size reduction of the waste before transportation. Depending on the required weight and volume capacity of transport, ranges were split into low, medium, and high ranges in the proposed system. The low range corresponds to standard capacity of small trucks, whereas medium range corresponds to trucks with long trailers. 

Transportation distance is a key parameter of uncertainty for transportation cost as it is difficult to define the exact location of facilities during cost estimation. It is assumed that the low range does not exceed 200 km, whereas the medium range for transportation is expected to be between 150 and 400 km. Membership functions for transportation and dismantling cost input are described in Table 1. The levels and table format are constructed based on the work developed by [32].

### 2.2. Dismantling Cost

Dismantling is needed to separate and collect the waste before its size reduction at the recycling plant. The end-of-life waste from wind blades or aircraft components contains different materials other than CFRP such as metals, foams and adhesive, and steel [33]. These materials need to be separated from recyclable composite structures. In this study, it is assumed that this phase is dependent on two input variables such as the volume of waste, and labour intensity. Moreover, the dismantling cost is not universal for each industry. For example, dismantling wind turbines is relatively cheaper than cars or aircrafts (see Table 2). However, dismantling is labour and cost-intensive process that is usually neglected in the cost estimation of CFRP recycling.

It was almost impossible to estimate dismantling cost using general rules for all types of shapes and forms of CFRP waste. For this purpose, the ranges were defined based on reported cost amongst different industries.

### 2.3. Operational Cost

Operational cost is incurred during the day-to-day running of a recycling facility which is dependent on the chosen technique and its maintenance procedures [36]. In this study, it is assumed that operational cost depends on the energy consumption and maintenance cost of the chosen recycling technique [7]. It is evident that the mechanical recycling option has the lowest operational cost, whereas thermal and chemical methods are more expensive. The variation of the energy consumption of recycling techniques is a major uncertainty factor. Table 3 represents the recycling technique and reported energy consumption levels.

The membership functions for energy consumption levels were constructed around the values reported from the literature. The fuzzy ranges for maintenance cost (M) are defined in the same way as for capital cost since it is a common practice that the maintenance cost of any equipment depends on its initial investments [25].

### 2.4. Capital Cost

The investment cost for comparison between recycling techniques are listed in Table 4. The reported cost was adjusted to the current period using the Chemical Engineering Plant Cost Index (CEPCI) of 2020, which was then transformed to a recycling capacity rate (1000 tons/year) using the six-tenths rule. The formula for calculating the final cost used for the input table is shown below [25]:

(1) $$C_{d}=C_{r}{\left(\frac{d}{r}\right)}^{0.6}\frac{I_{2020}}{I_{i}}$$

where, $C_{d}$—capital cost of a plant for a capacity ton per year; $C_{r}$—reference capital cost of a plant from the literature; r—indicated capacity in the literature (tons/year); d—planned annual capacity (tons/year); $I_{2020}$—CEPCI index in 2020; $I_{i}\;$—CEPCI index for the year of a reference plant.

The values were normalized linearly using the MAX method, which has the formula below [43]:

(2) $$n_{ij}=\frac{r_{ij}}{r_{max}}$$

where, $n_{ij}$—normalized value; $r_{ij}$—corresponding value in the matrix; $r_{max}$—the maximum value in the column.

### 2.5. Fuzzy Ranges

**Figure 2:**
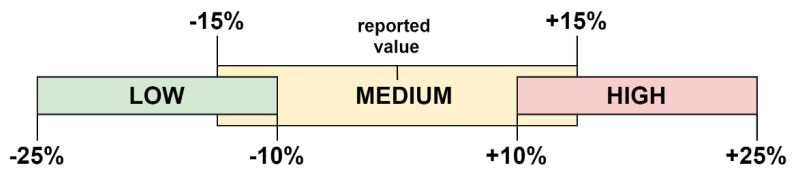
Adopted fuzzy ranges and corresponding deviations from the reported value.

The fuzzy ranges for linguistic expressions were constructed by allowing certain deviations from the reported values from the literature. The LOW range, for example, corresponds to −25% to −10% deviation from the provided parameter, whereas the MEDIUM range includes the deviation of 15% from the average. The HIGH range corresponds to 10% to 25% above the reported parameter. The ranges and their corresponding percentage deviations were presented in Figure 2. For instance, if the reported cost of dismantling in the aerospace industry is 0.54 EUR per kg, then the HIGH range for dismantling would be 0.594–0.675 EUR per kg. This rule does not indicate utility cost as the upper and lower limits were provided by the literature. Their ranges were defined by equally dividing the difference between upper and lower limits. 

**Figure 3:**
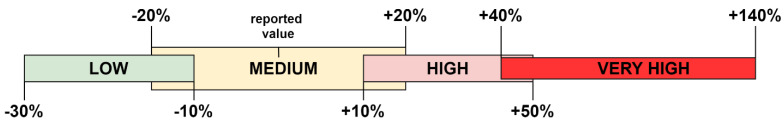
Adopted ranges for output cost.

The output cost ranges were defined by adjusting them according to outputs of other studies [7,24,25]. For output cost ranges, it was decided to construct four ranges that would extend the possible outcomes based on provided rules mention in Section 3.2. Figure 3 represents ranges for constructing output cost. 

For solvolysis in supercritical water technique (SCW), the range widths are narrower due to its already elevated reported cost. The VERY HIGH range for this technique is limited by +50% above the reported cost. The final values defining the matrix of output cost are represented in Table 5.

## 3. Development of the Fuzzy Logic Cost Modelling System

**Figure 4:**
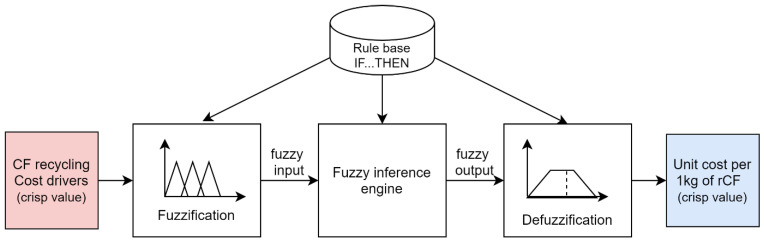
The overall strucutre of the fuzzy logic cost estimation.

A fuzzy logic approach is needed to address the uncertainty for unclear and ambiguous conditions [22]. In the case of recycling, various uncertainty factors can affect the final cost of recycling. For example, different energy consumption levels have been reported within the same process, which, in turn, can affect the accuracy of the final utility cost. In this case, IF–THEN fuzzy rules are helpful to handle the situation [22]. The final cost of the recycling process is estimated by the hierarchical structured fuzzy inference engines as shown in Figure 1. The overall framework of the proposed fuzzy assessment is illustrated in Figure 4. The first activity in the system development process was the identification of the necessary input and output parameters for the proposed fuzzy-logic system.

### 3.1. Fuzzification

Each inference engine computes one of the main five components of the final cost such as transportation, dismantling, operational, and capital cost, and annual quantity. Each inference engine follows the heuristic rules base of IF–THEN format which comprise the total cost at the end of the process. 

**Figure 5:**
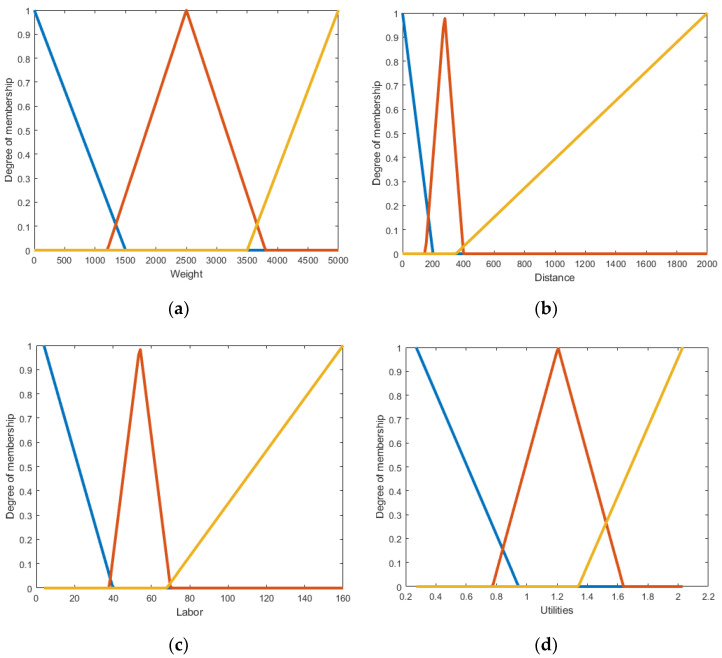
Membership functions of input parameters—(**a**) weight; (**b**) distance; (**c**) labor; (**d**) utilities. Fuzzy sets colour key: blue—low; red—medium; yellow—high; and purple line—very high.

**Figure 6:**
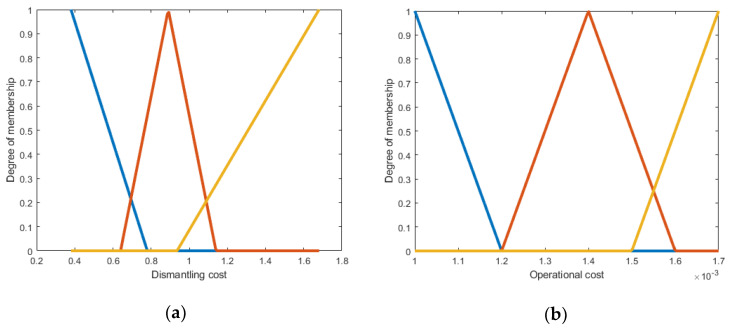

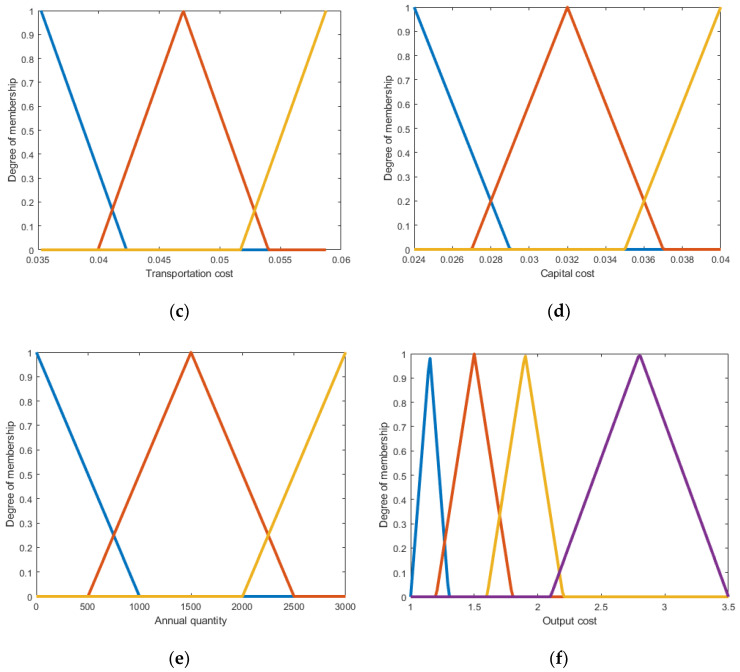
Membership functions of output cost and second-stage fuzzy inputs—(**a**) dismantling cost; (**b**) operational cost; (**c**) transportation cost; (**d**) capital cost; (**e**) annual quantity; (**f**) output cost. Fuzzy sets colour key: blue—low; red—medium; yellow—high; and purple line—very high.

Membership functions can be polygonal with the different number of hedges; however, in the proposed model triangular and trapezoidal membership functions were used due to their computational efficiency [46]. Moreover, membership functions were designed in a manner that ranges overlapped on the boundaries denoting the uncertainties there. The example of membership functions is shown in Figure 5a-d, where the x-axis shows low, medium, and high ranges as denoted in Section 2.1, while the y-axis shows the membership between 0 and 1. According to this triangular membership function, the system determines the ranges of output cost that correspond to user inputs for weight, size, distance etc. Similarly, membership functions for output cost are depicted in Figure 6a–f.

### 3.2. Fuzzy Inference

IF–THEN rules are based on the direct or indirect relationships between inference engines and principles that determine the total cost of recycling [32]. Knowledge bases are used to determine the components of total cost from several inputs collected from other knowledge bases. Moreover, the output of inference engines acts as inputs for other engines, and finally, the OUTPUT cost is computed from five main components. In addition, the first stage inputs may be used simultaneously in several inference engines, for example, weight and size of CFRP waste are simultaneously used inputs to estimate transportation and dismantling cost. 

A fuzzy expert system is built based on heuristic rules. The rules for input/output relationships are denoted in words or phrases, while mathematically they are expressed as fuzzy sets. An example of IF–THEN rules used in the model are shown below:

If UTILITIES is *low* and MAINTENANCE cost is *high*, then the OPERATIONAL cost is *medium*;

If TRANSPORTATION cost is *low* and CAPITAL cost is *medium* and DISMANTLING cost is *high* and OPERATIONAL cost is *medium,* then OUTPUT cost is *medium*.

Mamdani’s fuzzy inference method, an embedded method in MATLAB, is applied to estimate the “intermediate” cost. This step is followed by a defuzzification process that translates the fuzzy values into numeric values of the total cost [32,47].

The overall number of required fuzzy rules is subject to the number of inputs for each fuzzy set. The developed model uses several cost drivers which depend on the users’ input parameters, therefore, when a couple or more cost drivers are combined to estimate composite cost, the information loss might occur. To avoid high inaccuracy in the results, more linguistic indicators were used for each category of inputs. If the inference engine has *n* inputs and each having *k* linguistic values, e.g., *low, medium, high, very high,* then there will be k*^n^* fuzzy rules for a given inference engine. Moreover, one cost driver can have *m* sets of ranges for each recycling method (PYROLYSIS (P), FLUIDISED BED (FB), SUPERCRITICAL WATER (SCW)) therefore the number of fuzzy sets for each inference engine will be 3*k^n^.* Hence, the number of fuzzy rules used in the model to predict the total cost of CFRP recycling using a particular method can be derived as annual quantity and transportation cost, capital cost and dismantling cost and operational cost, which results in 243 distinct fuzzy rules (Table 6).

The system allows users to select the cost drivers and adjust the fuzzy rules for the recycling method where the total cost is estimated. Table 6 provides the rule base for output cost for the pyrolysis process.

### 3.3. Defuzzification

Finally, the fuzzy output follows a defuzzification process where it turns into a crisp value. Here, the inverse transformation process takes place as in the fuzzification process crisp domain is mapped into the fuzzy domain [48]. 

There are many ways to perform defuzzification, for example, the centre of gravity (COG) or the centre of sums (COS) and, the most popular, the centre of area method (COA) [49]. COA method is also called the centroid method and it was used in this model. COA method is the default method in MATLAB, and it calculates the centre of the area of the fuzzy set and determines the corresponding crisp value [50].

## 4. System Application and Results of Fuzzy System

**Figure 7:**
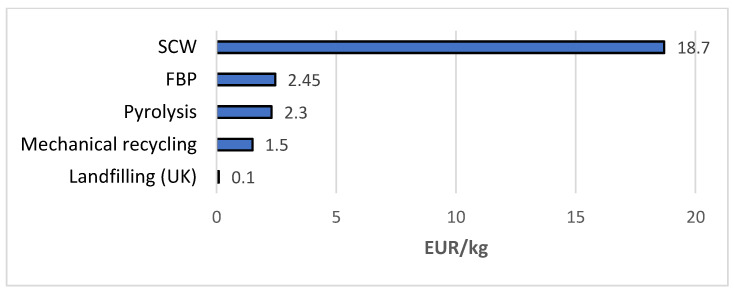
Comparison of recycling and landfilling cost (EUR/kg) generated by developed system.

Prior to assessing the impact of different factors on the total cost of recycling, the output costs of four recycling techniques were examined. This was done to compare the recycling cost with current landfilling cost. Assuming the default parameters to be 1000 tons of annual quantity and transportation distance to be 200 km, the comparison chart (Figure 7) shows the output recycling cost for CFRP recycling processes.

The cost of composite materials landfilling varies from 50 to 156 EUR per ton for most of the European countries [51]. The cost of landfilling in the UK was found to be about. 100 EUR per ton [51]. After comparing the cost, it is evident that landfilling is one of the most attractive options and will remain so for waste handlers if no special legal regulations are introduced. Additional incentives for recycling CFRP waste or increasing the cost of landfilling the waste could potentially help to shift towards recycling.

**Figure 8:**
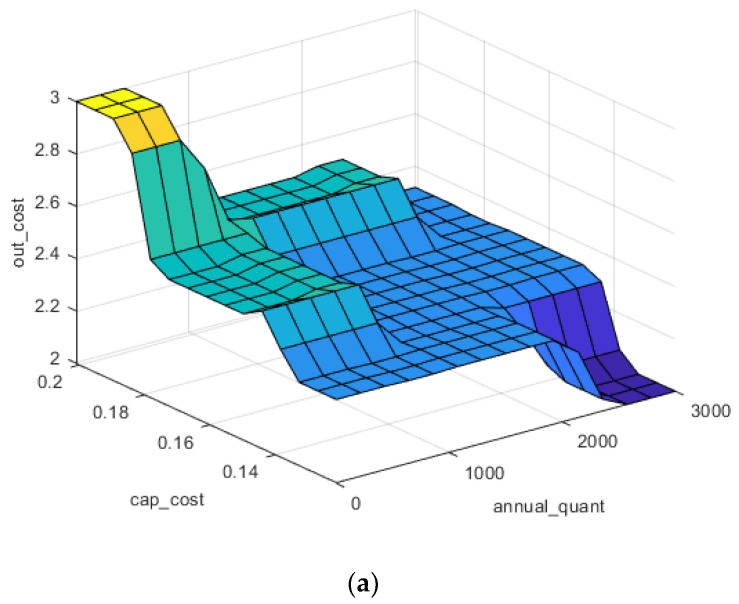

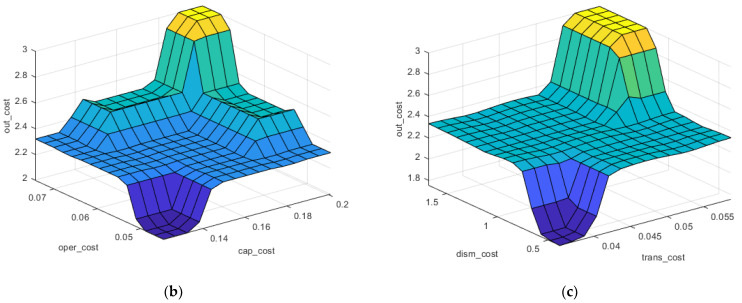
(**a**) Output cost levels related to the annual quantity and capital cost, (**b**) Output cost levels related to operational and capital cost, (**c**) Output cost levels related to transportation and dismantling cost.

Since the output cost ranges were predefined based on the reported cost found in the literature, the primary objective of this work, as discussed before, was to incorporate the uncertainty which is inherent in recycling processes. Figure 8a shows the contribution of the annual quantity of CFRP waste on the output cost for mechanical recycling. IF–THEN rules are constructed in a way that economy of scales applies for larger quantities which decreases the output cost at larger quantities recycled annually. In addition, the capital cost ranges chosen by the user also predefine the output cost which can be shown in Figure 8a. For instance, the higher capital cost at lower annual capacities results in significantly expensive output cost of recycling compared to lower capital cost with increased annual capacities.

Figure 8b represents the impact of operational cost and capital cost fluctuation on the output cost. Capital and operational cost both have significant influence on the output cost; however, coinciding ranges of two input parameters result in extremum cost levels (yellow and dark blue colours on the plot). Nevertheless, it is equally important to minimize capital investments at the initial planning phases as well as operational cost in order to have the lowest possible output cost for recycled products. Figure 8c shows the simultaneous effect of dismantling and transportation cost on output recycling cost. Not surprisingly, the same trend is noticeable: the extremums of low and high output cost are achievable only when operational and capital cost ranges coincide, e.g., output cost reach their lower range only when both parameters are at their lower ranges.

**Figure 9:**
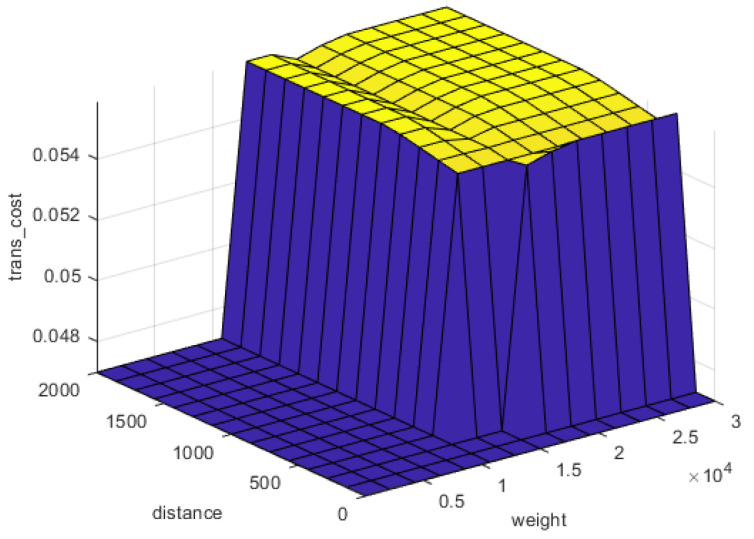
Transportation cost levels related to distance and weight.

The same charts with different inputs can be constructed by the system to determine the output cost of recycling at different levels of inputs or for other inference engines, i.e., transportation cost, dismantling cost. For instance, Figure 9 represents the relation of distance and weight of transported waste to the transportation cost. The further the distance between CFRP waste and recycling plant, the higher transportation coefficient will be applied for cost estimation which is illustrated by a yellow slope. For smaller weights up to one ton, the transportation coefficient shows low values, whereas larger weights leads to remarkably expensive transportation cost.

**Figure 10:**
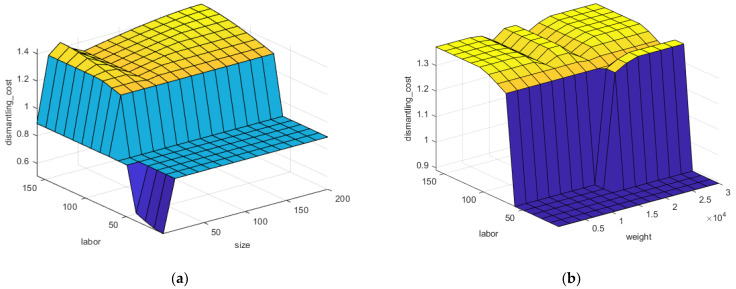
(**a**) Dismantling cost levels related to labour intensity and size (volume), (**b**) Dismantling cost levels related to labour and weight.

Figure 10 shows labour intensity, volume (size), weight, and their effect on dismantling cost for recycling CFRPs. Labour intensity is a critical factor that notably increases dismantling cost as illustrated in Figure 10a. For instance, cutting wind turbine blades during decommissioning is very labour-intensive process requiring special equipment such as wire saws set up on a vehicle [52]. As shown in Figure 10b, lighter components result in lower dismantling cost. However, it is clear from both charts that the major defining factor for dismantling cost is a labour intensity. Starting above 50 manhours for a component to be dismantled, dismantling cost is at its high range regardless the weight of a component. The more labour force requires for product disassembling the more it will lead to cost increase.

**Figure 11:**
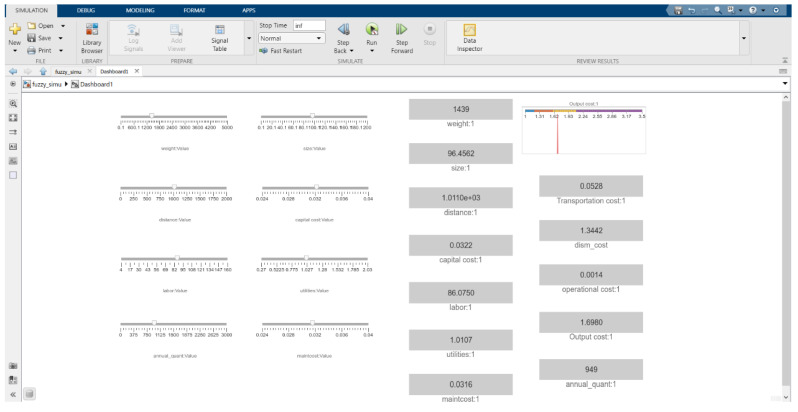
The user interface for selecting input parameters.

As the primary purpose of this model is to leverage uncertainty factors and determine the output cost of recycling CFRP waste, the final form of inputs and their variation is presented in slider form to easily adjust parameters for the user. The colour bar of the output cost shows in which range (low to very high) it is located. For instance, the red colour represents the middle range of output cost, whereas the yellow colour corresponds to the high range. The user interface for selecting input parameters is depicted in Figure 11.

To assess the developed fuzzy logic system with its rules, different case scenarios were tested for its sensitivity to simultaneous variation of inputs. Table 7 demonstrates eight random cases with different inputs for recycling techniques.

All the input parameters including the technique-dependent inputs and waste characteristics were adjusted and tested for different results. The ranges for technique-based inputs are indicated and chosen values are the average values within the chosen range. For instance, MEDIUM capital cost value for mechanical recycling is 0.032 (see Table 4). In addition, different maintenance and utility consumption levels were chosen to see the effect of operating cost. Overall, the fuzzy rules allow yielding results that incorporate all uncertainty factors considered in this work. The results demonstrate that the system is dependent on all inputs and the change in any input may affect the result. However, several statements could describe the behaviour of the whole fuzzy system and indicate points advised to be elaborated upon:

1. As the weight of five main inputs (Q, CC, TC, OC, and DC (see Figure 1 for notation)) for the output cost is equal (based on developed rules), the change of two or more of these inputs has a significant impact. For example, it is evident that the higher capital cost in combination with the higher operational cost (Maintenance + Utility) lead to the resultant highest cost.
2. Single inputs (CC, Q) without the first-level inputs have the highest effect on the output cost. This is clear from cases 7 and 8, where the increased annual quantity with similar other inputs decreased the output cost to almost 10%.
3. Other input parameters for dismantling and transportation cost acting together become a cost-increasing factor. For instance, comparing cases 2 and 3, even though capital cost were lower for case 2 than for case 3, the increased input parameters for waste characteristics (labour intensity and size) yielded results of more expensive output cost. Labour-intensive large waste components require additional cost for dismantling and transportation.
4. The accuracy of the system’s results is dependent on the chosen ranges for output cost and input parameters. The availability of data and expert knowledge is a critical factor for the correct implementation of the model. Ranges for output cost should be adjusted for the chosen market and country.

It should be noted that the values considered in this study may not depict the precise values in the current recycling market. The study’s limitations are inherent in fuzzy logic’s nature, which is a dependency on the expert knowledge and provided inputs. All cases considered are hypothetical and input values were extracted from the literature. Nevertheless, from this work, it is clear that single-point estimates are not capable of expressing the actual recycling cost for composite materials with their different characteristics and process parameters. The methodology developed in this study shows that the fuzzy logic approach is capable of working with these uncertainties.

## 5. Conclusions

The aim of this study was to develop a fuzzy logic-based system to estimate the recycling cost of CFRP waste taking into consideration all relevant uncertainties and inaccuracies. Recycling processes such as mechanical recycling, pyrolysis, fluidized bed process, and supercritical water were included in this work. The cost estimation system has employed heuristic rules in the form of IF–THEN rule. By applying the fuzzy theory, the ranges were constructed for imprecise cost drivers of recycling processes, and the Mamdani’s fuzzy inference system was implemented in MATLAB. The ranges developed are unique for every recycling method and CFRP waste origin. The fuzzy sets were constructed at the whole CFRP recycling cost structure including transportation, dismantling, operational, and capital cost. It is found that the uncertainty in capital cost, the planned annual capacity, and operational cost may result in considerable deviations of the final output cost of recycling. Transportation and dismantling cost as well at the same extent may affect the final cost of CFRP waste treatment. Moving towards the commercialization trend of CFRP recycling, stakeholders of CFRP market may find these results important. Specifically, the proposed methodology can be useful for end-of-life waste holders or recycling investors to evaluate the effects of uncertain elements in the cost structure of recycling and make informed decisions in the field. The study addresses the research gap amongst studies on cost modelling of recycling CFRP waste by considering waste parameters and quantifying the impacts of uncertainties in recycling processes. The illustrative case scenarios and a user-friendly interface helps managers to answer what-if scenarios promptly without requiring any deep quantitative knowledge. Future studies could explore this issue by investigating possible applications of recycled CFRPs in specific industries and their economic feasibility.

## Figures and Tables

**Table 1:** Membership functions of input variables for transportation and dismantling cost.

Input Variable	Level	Range
	Low	0–200
Transportation distance (km)	Medium	150–400
	High	350–2000
	Low	1000–12,600
Weight (Wind Turbine blades) (kg)	Medium	12,000–16,000
	High	15,400–30,000
	Low	0.1–10
Volume (m^3^)	Medium	8–50
	High	45–200
	Low	4–40
Labour intensity (manhours)	Medium	38–70
	High	68–160

**Table 2:** Dismantling cost in different industries.

Industry	Cost (EUR/kg)	References
Aerospace	0.54	[34]
Automotive	1.53	[24]
Wind turbine	0.42	[35]

**Table 3:** Energy consumption levels of different recycling techniques.

Recycling Technique	Energy Consumption (MJ/kg)	References
Mechanical recycling	0.27 (150 kg/h) 2.03 (10 kg/h)	[37]
Pyrolysis	2.8 30	[38] [39]
Fluidized bed process	6 (at 12 kg/h·m^2^ feed rate)	[40]
Solvolysis	63–91	[41,42]

**Table 4:** Capital investments for recycling techniques.

Technique	Capital Investment According to the Literature	Adjusted Capital Cost Up-to-Date	Capital Cost at a Capacity of 1000 Tons/Year	Normalized Values
Pyrolysis	10,000,000 EUR for a capacity of avg. 50,000 tons per year [7]	10,188,034 EUR for a capacity of avg. 50,000 tons per year	974,335 EUR	0.16
Mechanical	200,000 EUR for a capacity of 4000 tons per year [44] (only shredder)	425,714 EUR for a capacity of 4000 tons per year (a hammer miller included)	185,303 EUR	0.03
Fluidized bed	4,100,000 EUR for a capacity of 1000 tons per year [25]	4,379,211 EUR for a capacity of 1000 tons per year	4,379,211 EUR	0.72
Supercritical Water	5,770,000 EUR for a capacity of 150 kg per hour [45]	6,065,115 EUR for a capacity of 150 kg per hour	6,065,115 EUR	1

**Table 5:** Recycling techniques and fuzzy ranges for output cost.

Output (Level 2)	Level	MECHANICAL		PYROLYSIS		FBP		SCW	
Cost	LOW	1	1.3	1.6	2.1	1.7	2.2	14	16.8
	MEDIUM	1.2	1.8	1.8	2.8	2	2.9	15.9	21.5
	HIGH	1.6	2.2	2.5	3.5	2.7	3.7	20.6	23.4
	VERY HIGH	2.1	3.5	3.2	5.5	3.4	5.9	22.5	28.1

**Table 6:** CFRP recycling output cost rules.

Rule Rk	If ANNUAL QUANTITY Is	If TRANSPORTATION COST Is	If CAPITAL COST Is	If DISMANTLING COST Is	If OPERATIONAL COST Is	Then OUTPUT COST Is
R1	*low*	*low*	*low*	*low*	*low*	*low*
R2	*low*	*low*	*low*	*low*	*medium*	*low*
R3	*low*	*low*	*low*	*low*	*high*	*medium*
R4	*low*	*low*	*low*	*medium*	*low*	*low*
R5	*low*	*low*	*low*	*medium*	*medium*	*medium*
…						
R239	*high*	*high*	*high*	*medium*	*medium*	*medium*
R240	*high*	*high*	*high*	*medium*	*high*	*high*
R241	*high*	*high*	*high*	*high*	*low*	*medium*
R242	*high*	*high*	*high*	*high*	*medium*	*high*
R243	*high*	*high*	*high*	*high*	*high*	*high*

**Table 7:** Different case scenarios and predicted output cost for recycling CFRPs.

CASE No.	INPUTS								OUTPUT Cost (EUR/kg)			
	Recycling Technique Parameters			Waste Characteristics								
	Capital Cost	Maintenance	Utility Level	Weight, kg	Size, m^3^	Labour Intensity, Manhours	Distance, km	Annual Quantity, tons	Mechanical	Pyrolysis	FB	SCW
1	MEDIUM	LOW	LOW	100	10	5	250	1500	1.15	1.7807	1.88	18.6992
2	MEDIUM	LOW	LOW	50	1	10	200	1500	1.3	1.7751	1.8748	15.0128
3	LOW	MEDIUM	LOW	2500	50	80	500	2900	1.5	2.3227	2.4501	18.6998
4	MEDIUM	MEDIUM	LOW	1200	2	40	1000	1500	1.5	2.3281	2.4502	18.6996
5	HIGH	MEDIUM	LOW	500	50	70	100	500	1.9	2.5768	3.2	21.9997
6	HIGH	HIGH	MEDIUM	1500	5	30	1500	1250	1.5	2.3248	3.55	22
7	HIGH	HIGH	HIGH	4000	8	70	1000	500	2.8	4.5398	4.8242	24.6484
8	HIGH	HIGH	HIGH	4000	15	80	1000	2250	2.53	4.0954	4.2743	24.3502

## Data Availability

The relevant data are all included in the paper.
